# Nano Pt-decorated transparent solution-processed oxide semiconductor sensor with ppm detection capability†

**DOI:** 10.1039/c8ra09917k

**Published:** 2019-02-20

**Authors:** Jingu Kang, Kyung-Tae Kim, Seoung-Pil Jeon, Antonio Facchetti, Jaekyun Kim, Sung Kyu Park

**Affiliations:** School of Electrical and Electronic Engineering, Chung-Ang University Seoul 06974 Republic of Korea skpark@cau.ac.kr; Department of Chemistry and the Materials Research Center and the Argonne-Northwestern Solar Energy Research Center, Northwestern University Evanston Illinois 60208 USA; Flexterra Inc. Skokie Illinois 60077 USA; Department of Photonics and Nanoelectronics, Hanyang University Ansan Gyeonggi-do 15588 Republic of Korea jaekyunkim@hanyang.ac.kr

## Abstract

In this study, we fabricated a transparent Pt-decorated indium gallium zinc oxide (IGZO) thin film based on a solution process to demonstrate a portable, low-cost volatile organic compound (VOC) based real-time monitoring system with the detection capability at as low as 1 ppm. The Pt/IGZO sensor shows remarkable response characteristics upon exposure of isobutylene (2-methylpropene) gas down to 1 ppm while also maintaining the reliability and reproducibility of the sensing capability, which is almost comparable to a commercial VOC sensor based on a photoionization detector (PID) method. For 1 ppm of isobutylene gas, the response and recovery time of the sensor estimated were as low as 25 s (*S*_90_) and 80 s (*R*_90_), respectively. The catalytic activity of Pt nanoparticles on an IGZO nano-thin film plays a key role in drastically enhancing the sensitivity and dynamic response behaviour of the VOC sensor. Furthermore, the solution-processed IGZO thin film decorated with Pt nanoparticles also represents a highly transparent (in visible region, ∼90%) and low-cost fabrication platform, thereby, facilitating the optical visibility and disposability for future applications in the field of electronics. Therefore, we believe that the nano-Pt/IGZO hybrid material for VOC sensor developed by us will pave a way to detect any harmful chemical gases and VOCs in various environments.

## Abstract 2

A transparent VOC sensor based on a solution-processed IGZO for application in real-time VOC monitoring system.

## Graphical abstract

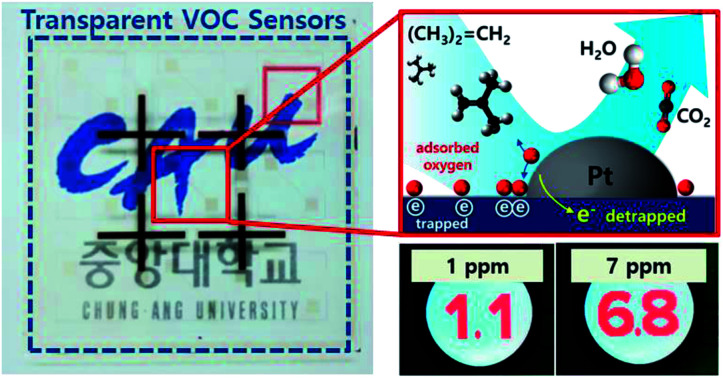

## Introduction

Rapid civilization and technological advances cause variety of environmental problems all over the world; air, water, and, land pollution are being intensified even now. Especially, the air quality of the house, school, and workplace is the top priority issue for human health. There are many volatile organic compounds (VOCs) in the indoor atmosphere such as, formaldehyde, benzene, and toluene that easily evaporate at ordinary room temperature. They are considered as primary indoor air pollutants.^1–3^ The excessive exposure to VOCs can give rise to both short- and long-term effects on various aspects of human health, from mild symptoms with respect to skin, eyes, respiratory system, or headache to damage of kidney, liver, or central nervous system.^3–5^ Therefore, the emission and the level of VOCs should be detected and monitored by a monitoring system in real-time for taking proper countermeasures and assure safety.

In the industrial field, various technologies have been employed to evaluate the concentrations of VOCs in air, for example, gas chromatography-mass spectroscopy (GC-MS), photoionization detector (PID), and chemiresistor.^6–10^ GC-MS is a traditional analytical method requiring samples of target gases, and thus have restrictions such as high-cost and non-real-time measurement. PIDs are commonly used in commercialized VOC detection systems, especially, portable equipments, showing a rapid detection and recovery, but it is also very expensive. Chemiresistors based on metal oxides (MO) are widely used in commercialized detection systems. The chemiresistors have simple structures and show good sensing characteristics, which can attract more attention and interest for application in real-time monitoring systems showing a specific concentration of a target VOC.^7,11^

Particularly, for detecting various VOCs, a variety of metal-oxide materials such as ZnO, SnO_2_, and TiO_2_ have been actively explored and utilized as a sensing layer due to their electrical properties, high sensitivity, and fast response.^12^ The MO sensors exhibit resistance changes due to the redox reaction between gas molecules and adsorbed-oxygen molecules at the sensing layer. For instance, while the reducing gas molecules react with the adsorbed-oxygen molecules, the electrons are released into the MO layer and are involved in charge transport, reducing the resistance of the MO layer.^13^

In addition, the sensitivity of the polycrystalline- or nano-structured-MO sensors is mainly dependent on the width of the depletion region between the MO grains. Therefore, diverse types of MO nano-structures including nanowires, meso- and micropores, and nano-spheres are intensively studied in an attempt to maximize the surface-to-volume-ratio, which facilitates the adsorption of gas molecules. However, such technologies also have restrictions such as requirement of complex synthesis processes or high temperature processes exceeding 500 °C for the grain growth.^14,15^

To overcome the limitations, a solution-processed MO semiconductor as the sensing layer is a great candidate for the fabrication of a VOC sensor involving a cost-effective and a relatively low-temperature process (<500 °C), and a simple synthesis of the precursor solution.^16^ Furthermore, a solution-processed MO sensor is suitable for a low-cost replacement in a gas monitoring system. However, realization of a prototype VOC monitoring system based on a solution-processed indium gallium zinc oxide (IGZO) sensor has not yet been reported.

In this study, we fabricated a transparent solution-processed IGZO gas sensor using a noble metal in catalytic amounts for the real-time VOC monitoring system. We investigated the response and recovery characteristics of the MO sensor for isobutylene gas, which is a calibration gas for PIDs. In addition, the sensing test was repeatedly conducted with various concentrations of isobutylene from 100 ppm down to 1 ppm to obtain the reliability and reproducibility of the test. Furthermore, we demonstrated a prototype of a portable VOC monitoring system, which can be operated by a portable power bank. The portable VOC monitoring system detected specific concentrations of isobutylene in real-time and presented it through a liquid crystal display (LCD) on the circuit board.

## Experimental

### Sensing device fabrication

The precursor solution of IGZO was composed of 0.085 M of indium nitrate hydrate (In(NO_3_) _3_·*x*H_2_O), 0.0125 M of gallium nitrate hydrate (Ga(NO_3_) _3_·*x*H_2_O), and 0.0275 M of zinc acetate dihydrate (Zn(CH_3_CO_2_)_2_·2H_2_O) in 2-methoxyethanol (2-ME), and then, the solution was stirred at 75 °C for 12 h at least.

A glass was used as the substrate (Eagle 2000, Corning) for the Pt/IGZO sensor and was cleaned by sonication in acetone and isopropyl alcohol for 10 minutes each. In order to get rid of organic residues and form the hydrophilic surface, the substrate was treated by oxygen plasma in a reactive ion etching (RIE) system. The precursor solution was dispersed onto the substrate and was spun at 3000 rpm for 20 s by a spin coater. Afterward, hot plate annealing was carried out in two steps: firstly, at 200 °C for 10 minutes; secondly, at 350 °C for 1 h. The thickness of the IGZO thin film was about 10 nm, and the thin film was patterned by conventional photolithography, which was estimated by scanning the patterned IGZO using an atomic force microscopy (AFM), (Fig. S1, ESI†). To prepare interdigitated electrodes, 100 nm of indium zinc oxide (IZO) was deposited by RF-sputtering and lifted off. IZO was chosen as the metal contact to IGZO due to its optical transparency and its similar work function compared to that of IGZO. The work functions of IZO and IGZO are 4.09 eV and 4.25 eV, respectively.^17^ The width and the length of final IGZO devices are 10 mm and 50 μm, respectively.

**Fig. 1:**
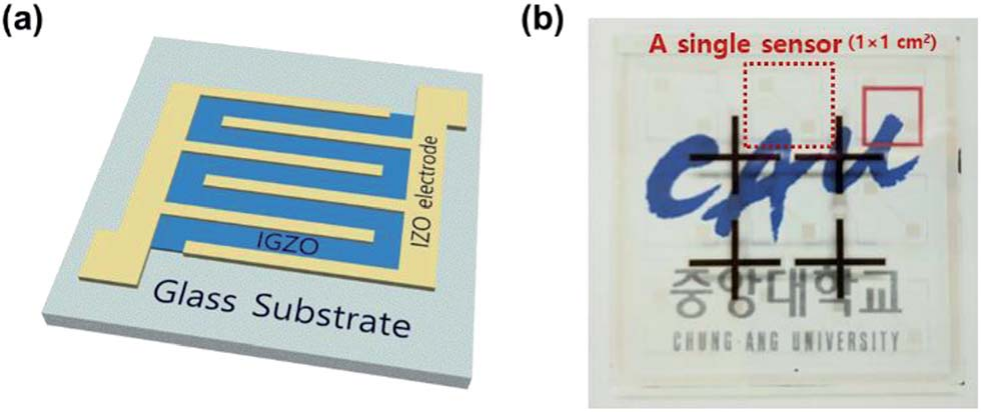
(a) The schematic of the final device structure magnified the sensing layer (1 × 1 mm^2^). The width and length are 10 mm and 50 μm, respectively. (b) The transparent Pt/IGZO gas sensors on a glass substrate (5 × 5 cm^2^). The sample size of the single Pt/IGZO is 1 × 1 cm^2^ (red dot square).

To introduce a catalytic metal on the MO surface only, Pt was deposited by an ion-coater with 3 mA of ionization current for 50 s (SPT-20, COXEM) with a screen mask. The final device structure is depicted in the inset of Fig. 1(a). The dimension of the sensing layer is 1 × 1 mm^2^ on a glass substrate (1 × 1 cm^2^), as shown in Fig. 1(b). The surface morphology and the film thickness of the MO sensing layer were analyzed by AFM (NX-10, Park systems). X-ray diffraction (XRD) spectra were obtained using D8-Advance (Bruker ASX) X-ray diffractometer. Field emission scanning electron microscopy (FE-SEM) images and energy dispersive spectroscopy (EDS) spectra were obtained using the Regulus 8230 (Hitachi) ultra-high resolution electron microscope. The transmittance spectrum of the transparent MO gas sensor was measured by a UV/Vis spectrophotometer (LAMBDA 35, PerkinElmer).

### Gas sensing measurement

**Fig. 2:**
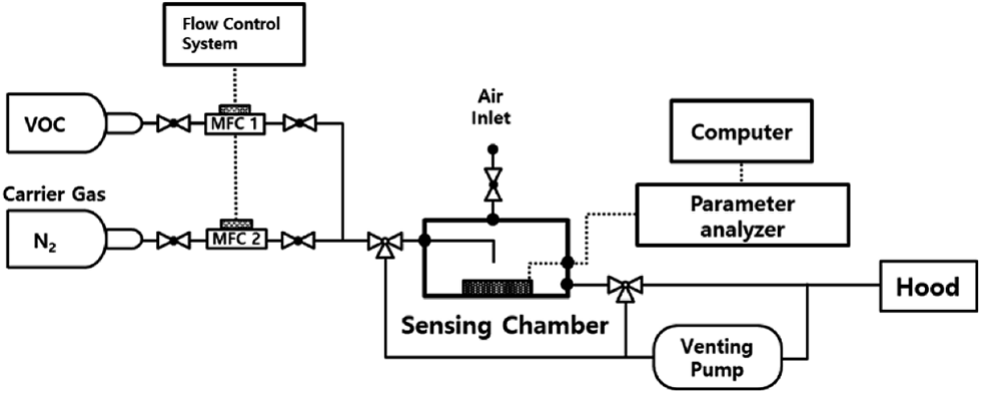
The block diagram of the gas sensing test system with a parameter analyzer and MFC control system.

The gas sensing properties were measured using a semiconductor parameter analyzer (4155C, Agilent) in a homemade dark-box with a gas flow control system, as shown in Fig. 2. The Pt/IGZO sensor was placed beneath the outlet of the final gas tube at a distance of 1 cm, with a direct exposure to the VOC. The sensing characteristics were measured at 160 °C. The final concentration of isobutylene was controlled by dilution with pure nitrogen, which was additionally checked by a commercial VOC detector based on PID (Tiger, Ion Science). The flow rate of the final concentration was 500 sccm, which was controlled by mass flow controllers (MFCs), and the sequential on/off switching of VOC was automatically regulated by a flow control system. The sensing chamber was kept open through the air inlet and the venting line. Also, the venting pump was always on to circulate air in the sensing chamber.

## Results and discussion

To identify the characteristics of the sensing layer, we analyzed the film phase of the pristine IGZO and Pt/IGZO as shown in XRD spectra (Fig. S2, ESI†). The pristine IGZO film shows the formation of an amorphous phase, which suggests that the amorphous film annealed at 350 °C can support the stable and reliable sensing properties as the MO sensor operates at 160 °C. Moreover, Pt/IGZO film shows the same spectrum as that of the pristine one due to the very thin film of Pt.^18^

**Fig. 3:**
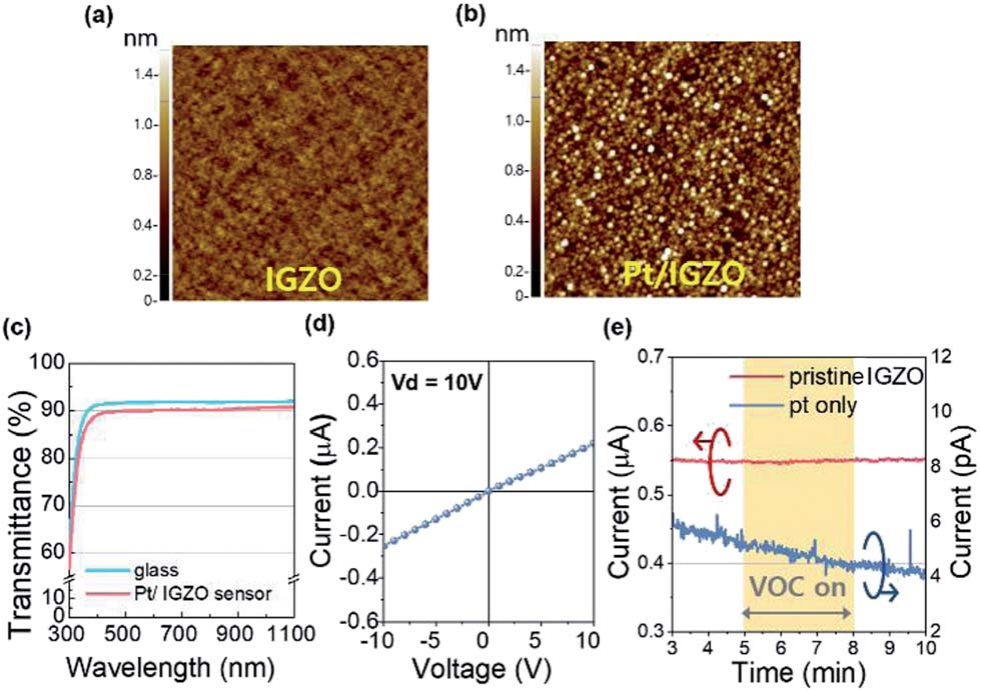
The AFM images of the surface morphologies for (a) the pristine IGZO (RMS = 0.12) and (b) Pt/IGZO (RMS = 0.49). The scale of the AFM images is 1 × 1 μm^2^. (c) The transmittance spectrum of a glass substrate (blue solid) and the Pt/IGZO gas sensor (red solid). (d) *I*–*V* characteristics of the Pt/IGZO sensor. (e) The current level of the pristine IGZO (red solid line, left-*y*-axis) and the Pt layer (blue solid line, right-*y*-axis). The exposure time of isobutylene gas is 3 min.

We examined the surface morphology to clarify the homogeneous deposition of the nano-structured Pt upon IGZO. Fig. 3(a) and (b) are AFM images of the surface morphologies for the pristine IGZO and Pt decorated IGZO, respectively. The root mean square (RMS) roughness of the pristine IGZO is 0.12 nm and that of Pt/IGZO is 0.49 nm. The RMS roughness of the Pt/IGZO increased about 4 times than that of the pristine one due to the nano-scale grains of Pt (*D*: 20–30 nm), as observed in Fig. 3(b). Furthermore, we performed FE-SEM and EDS analysis, as shown in Fig. S3.† The FE-SEM image of Pt/IGZO also presents Pt grains, which is consistent with the AFM data, and the EDS spectrum of Pt/IGZO evidently reveals the prominent peak of Pt. These results demonstrate that the amorphous IGZO film was obtained without any cracks and the Pt decoration upon IGZO was uniformly achieved.

Also, we investigated the transmittance of the Pt/IGZO gas sensor as shown in Fig. 3(c). The Pt/IGZO gas sensor exhibits a high optical transmittance of 90% in the visible light region, compatible with a glass substrate, which is attributed to the large band gap of IGZO (3.2 eV) and IZO (2.9 eV) and the small fraction of the sensing layer in a single sensor substrate. This transparent Pt/IGZO gas sensor can be applied in diverse transparent materials, such as windows, for the detection of indoor or outdoor VOC as shown in Fig. 1(b).

Before investigating the gas sensing properties, we measured the *I*–*V* characteristics of a pristine IGZO film to examine the contact property between the interdigitated electrodes of IZO and the sensing layer of IGZO. As shown in Fig. 3(d), the linear *I*–*V* characteristic was observed within the operating voltage from −10 V to 10 V, demonstrating the ohmic contact between the electrodes and the sensing layer. Also, we investigated the gas response of the pristine IGZO under the exposure of 100 ppm of isobutylene gas for 3 min. Fig. 3(e) (left *y*-axis) indicates that the pristine IGZO cannot detect the isobutylene gas, which can be attributed to the insufficient redox interaction of adsorbed oxygen species with gas molecules at the operating temperature of 160 °C.^19^ Hence, we employed platinum for rendering the VOC detection of the IGZO sensor highly sensitive and reliable.

Noble metals are generally used as catalysts for facilitating redox reaction, particularly, in gas sensors.^20–22^ Although many studies report complex chemical compositions with noble metals and sensing materials in synthesis steps, we simply use an ion coater to deposit the catalytic noble metal onto the IGZO surface because the redox reaction between the gas molecules and the sensing layer predominantly occurs at the MO surface.^13,20–22^ As a result, the Pt-decorated IGZO sensor shows excellent responses to the VOC, as discussed below.

Moreover, in order to ensure that the charge transport occur primarily through the MO layer during the operation of the sensor, we measured the current response as a function of time for the Pt layer only, which was formed with the same deposition condition of the Pt/IGZO sensor. As shown in Fig. 3(e) (right *y*-axis), the current level of the Pt layer was observed to be as low as ambient or equipment noise level, showing no response to the VOC.

Accordingly, since the nano-grains of Pt on the MO sensing layer act as a catalyst and rarely provide a direct charge transport, solely the surface-to-volume ratio of the Pt/IGZO is enhanced without hindering the charge transport by the grain boundaries.

The sensing mechanism of the MO sensor has been enormously reported and well established.^13,23^ The sensitivity of MO sensors is traditionally defined by the change in electrical conductivity that can be ascribed to the reaction between the gas molecules and the surface-adsorbed oxygen species, while, fundamentally, the adsorption and desorption of oxygen species depend on the reaction temperature.^13^ Oxygen species in air are first physically and then chemically adsorbed onto the MO surface, leading to a wider space-charge region at the surface due to electron withdrawal by the adsorbed-oxygen species. As a result, the resistance of the MO sensing layer is increased due to the decrease in the carrier concentration within the charge transport path. When a reducing gas reaches the surface, the redox reaction takes place between the surface-adsorbed oxygen species and the reducing gas molecules, releasing electrons into the MO layer. Consequently, this reduces the space-charge region and the resistance of the MO layer.

The types of surface-adsorbed oxygen species varies with temperature, *viz.*, O_2_^−^, O^2−^, and O^−^. In general, the atomic oxygen species (O^−^) begins to dominate above 150 °C by acquiring electrons from the MO layer, as described below.^13,19,24^

1 O_2_ (ads.) + e^−^ = O_2_^−^

2 O_2_^−^ + e^−^ = 2O^−^

**Fig. 4:**
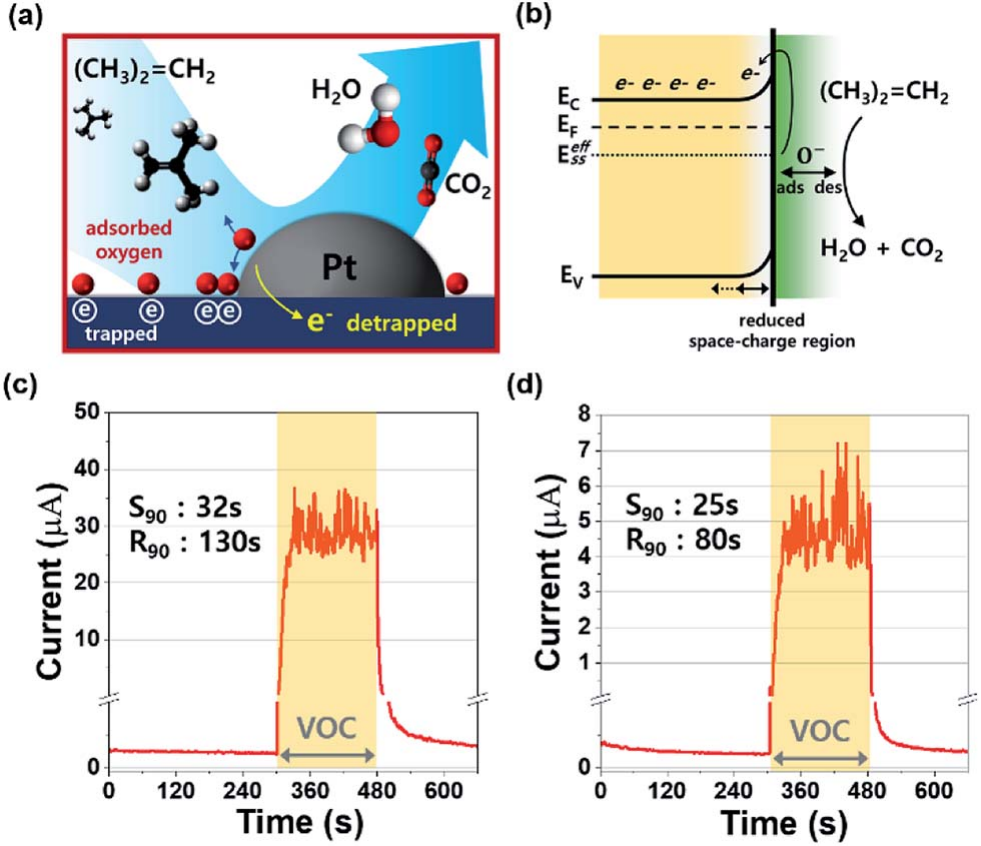
(a) The illustration of the sensing mechanism between isobutylene molecules and Pt/IGZO film. More atomic-oxygen species reside near Pt particles, withdrawing electrons from the IGZO film. When the oxygen species are desorbed, the trapped electrons are released to the IGZO film. (b) The energy band diagram when isobutylene gas molecules react with adsorbed oxygen species. (c) and (d) The response and recovery characteristics for isobutylene detection of (c) 100 ppm and (d) 1 ppm.

In this study, the adsorption and desorption of oxygen species take place at 160 °C, and the atomic form (O^−^) is more reactive to the MO surface than the others (O_2_^−^ and O^2−^). Therefore, the atomic form of oxygen (O^−^) dominates at the surface, reacting with the gas molecules in the periphery of the sensing surface. Fig. 4(a) depicts the sensing mechanism of the Pt/IGZO sensor for isobutylene gas. Since the sensing chamber was kept open, the oxygen species are readily adsorbed and bonded to the IGZO surface. While the sensor is on, the atomic oxygen species dissociate and form O^−^, and the rest of the oxygen species desorb from the surface. The catalytic noble metal enables the atomic oxygens to reside near the catalyst particle, which facilitates the dissociation of oxygen species and the redox reaction at the MO surface.^23^ In addition, an effective surface state (*E*^eff^_SS_) was formed by surface-adsorbed O^−^, in which electrons are trapped creating a wide space-charge layer.^25^ When the isobutylene gas is introduced to the Pt/IGZO surface, the redox reaction occurs between the VOC molecules and the MO surface, releasing electrons to the conduction path as described below.^26^

3 (CH_3_)_2_=CH_2_ + 12O^−^ (ads.) ↔ 4H_2_O + 4CO_2_ + 12e^−^

Consequently, after the IGZO surface is exposed to the isobutylene gas, the widened space-charge region is reduced due to the detrapped electrons from the oxygen species, making the conductivity of the IGZO layer high. When the isobutylene injection is stopped, the Pt/IGZO sensor returns to the initial state by forming oxygen species at the surface, showing a lowered conductivity.

For the real-time VOC monitoring system, the precisely quantified steps for each concentration of the gas are essentially required to convert the electrical analog signal to discrete concentration units like parts per million (ppm). Accordingly, the response and recovery points for each concentration should be defined at their saturation levels. Therefore, we investigated the gas response and recovery characteristics for 100 ppm and 1 ppm of isobutylene gas as shown in Fig. 4(b) and (c).

Prior to the exposure to isobutylene gas, the Pt/IGZO sensor was initialized under pure nitrogen for 5 minutes. Subsequently, the sensor was exposed to the 100 ppm or 1 ppm of the diluted isobutylene gas for 3 minutes, and then, the VOC was off. Fig. 4(b) shows the sensing characteristics towards the 100 ppm of isobutylene, in which, the current is rapidly increased and is saturated around 30 μA when the VOC is detected. For detecting 1 ppm of isobutylene, the current is quickly increased and is saturated around 4.5 μA when the VOC is detected, as shown in Fig. 4(c). In both these cases, the Pt/IGZO sensor demonstrates the outstanding response and recovery characteristics towards the isobutylene gas at as low as 1 ppm. In addition, the current fluctuations are also observed due to the non-ideal flow of the diluted gas, which imply that our Pt/IGZO sensor is highly sensitive to the variation of VOC concentration. In order to define the response time (*S*_90_) and the recovery time (*R*_90_) of the sensor, essentially a precise saturation current (or resistance) value is required when the sensor is under exposure of a target gas. In this case, however, it is difficult to define the saturation due to the instant response to the gas fluctuation. Therefore, we calculated the median with 30 points of the current, and then, we defined *S*_90_ and *R*_90_ by the standard procedure.^27–29^ As a result, the *S*_90_ and *R*_90_ for 100 ppm are 32 s and 130 s, respectively. For 1 ppm, the *S*_90_ and *R*_90_ are 25 s and 80 s, respectively. Given that gas sensors usually suffer from unsaturated response and insufficient recovery to their initial state, our VOC sensor shows the full saturated response and full recovery to the initial state, which is very beneficial for implanting into a real-time VOC monitoring system.

**Fig. 5:**
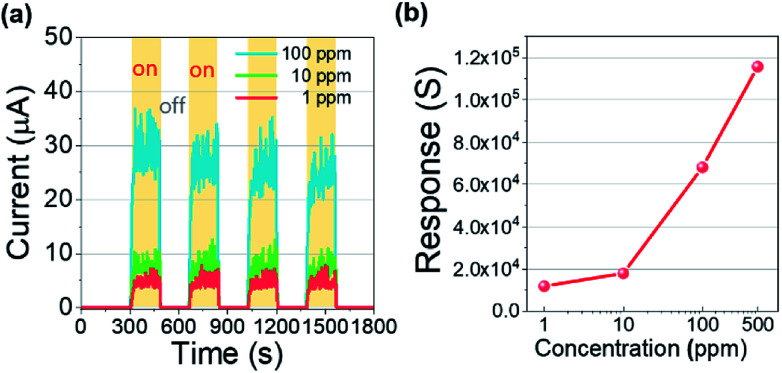
(a) The reliability and reproducibility of the VOC detection characteristics for 100, 10 and 1 ppm. The on/off interval is 3 minutes. (b) The response to isobutylene as a function of gas concentrations; 11,541; 11,611; 67,843; and 114,561 for 1, 10, 100, and 500 ppm, respectively. (*S* = *R*_0_/*R*_g_).

Moreover, a reliable response and recovery of a gas sensor are important requirements for the realization of a real-time VOC monitoring system. So, we additionally examined the reliability and reproducibility of the VOC response with a switching gas exposure as shown in Fig. 5(a). Each concentration of isobutylene was significantly and repeatedly detected, exhibiting the saturated current level and the immediate recovery for 100 ppm, 10 ppm, and 1 ppm. These excellent reliability and reproducibility of the VOC detection enable the analogue electrical signal to be quantified into discrete concentrations for establishing the VOC monitoring system. The responses of the sensor toward 500, 100, 10, and 1 ppm of isobutylene were calculated and plotted as shown in Fig. 5(b). The response (*S*) to the isobutylene was defined as *S* = *R*_0_/*R*_g_, where *R*_0_ and *R*_g_ are the resistances when the target gas was off and on, respectively.^30–32^ In addition, the *R*_g_ was extracted from the *S*_90_ point for each concentration. The Pt/IGZO sensor demonstrates the response to isobutylene gas as 11,541; 11,611; 67,843; and 114,561 for 1, 10, 100, and 500 ppm, respectively. These exceptional detection characteristics towards isobutylene gas reveal that Pt/IGZO sensor is adequate for a practical application.

**Fig. 6:**
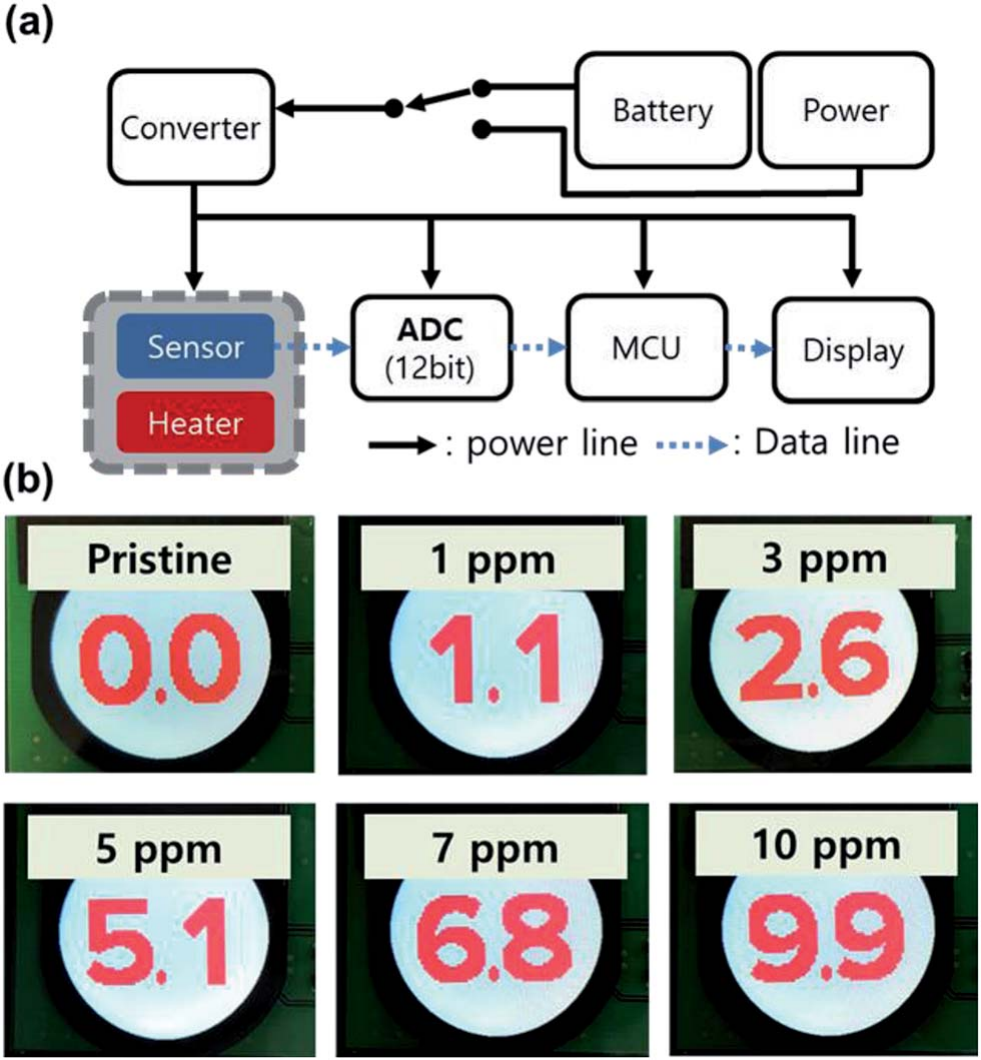
(a) The block diagram of the portable real-time VOC monitoring system that can be operated by a portable power bank. (b) The photographs of the monitoring result in real-time. The 100 steps of VOC concentrations are displayed with 0.1 resolution, including the calibration points; pristine, 1, 5, and 10 ppm.

Based on the Pt/IGZO gas sensor, we fabricated a prototype of a portable real-time VOC monitoring system as shown in the block diagram of Fig. 6(a). The sensor part consists of the Pt/IGZO sensor and an underlying home-made heater, and then, they were assembled and were wired upon a small socket, which is connected to a circuit board. The output signal of the sensor is input into an analogue-to-digital converter (ADC, 12-bit), and the digitized signal is transferred to a micro controller unit (MCU). In order to compensate the unexpected noise signal, the sensing data are collected and accumulated every 50 ms in the MCU to be calculated into an average value with the latest 20 points. Finally, the LCD panel of the VOC monitoring system displays the concentration of isobutylene gas for every second with the resolution of 0.1 ppm. The real-time VOC monitoring system can be operated by a portable power bank.

We determined a detection range from 0.0 to 9.9 ppm with intermediate concentration points at 1 and 5 ppm. Therefore, 4096 points of the ADC were divided into 4 step concentrations at 0, 1, 5 and 10 ppm. Prior to a real-time VOC monitoring test, the system was initially calibrated with the concentration-controlled isobutylene gas by a PID VOC detector for matching values between the ADC and the 4 steps of calibration points. Subsequently, we conducted a real-time VOC monitoring test with arbitrary gas concentrations, powered by a potable power bank. As shown in Fig. 6(b), when the portable VOC monitoring system was exposed to isobutylene of 1, 3, 5, 7, and 10 ppm, it successfully showed the specific concentrations of isobutylene gas as 1.1, 2.6, 5.1, 6.8, and 9.9 ppm, respectively. Furthermore, we examined the relative standard deviation (RSD) for each concentration, as summarized in Table S1 (ESI†). RSD values are 20.56%, 7.73%, 5.05%, 7.36% and, 3.43% for 1, 3, 5, 7, and 10 ppm of isobutylene, respectively. The RSD of 1 ppm is almost 3 times higher than that of 3 ppm due to the steady standard deviations regardless of the gas concentration. In the case of the detection for 3 and 7 ppm, lower values are obtained than those of the MFC-controlled concentrations, which can be attributed to the non-ideal linearity of the response from 1 to 5 ppm and from 5 to 10 ppm, respectively. However, the mismatch can easily be reduced by inserting calibration points. Accordingly, the prototype of the portable real-time VOC monitoring system can be used in practical situations.

## Conclusions

MO semiconductors are beneficial in sensor applications due to their electrical properties, high sensitivity, and fast response. In addition, solution-processed MO can realize a low-cost process based on a low temperature and a simple synthesis process. The transparent solution-processed sensing layer of IGZO was formed, and then, the catalytic Pt was deposited upon the IGZO layer. The Pt/IGZO gas sensor exhibits a high optical transmittance of 90% in the visible light region. The catalytic noble metal facilitates the redox reaction between isobutylene gas molecules and atomic oxygen species at the MO surface, leading to outstanding response and recovery characteristics. The Pt/IGZO sensor demonstrates 25 s of response time and 80 s of recovery time for 1 ppm of isobutylene. In addition, excellent reliability and reproducibility of detection properties were observed by repeating the sensing test.

Finally, the Pt/IGZO sensor was implanted as a portable real-time VOC monitoring system. Given that the sensor shows significant saturation signal for each concentration of isobutylene, the Pt/IGZO sensor is well-suited for a real-time detection system. The portable-real-time VOC monitoring system demonstrates an excellent detection property compared to that of a PID-based VOC detector.

## Conflicts of interest

There are no conflicts to declare.

## Supplementary Material

RA-009-C8RA09917K-s001
