# Supplementary material for: Nano Pt-decorated transparent solution-processed oxide semiconductor sensor with ppm detection capability
Source: RSC Adv. 2019 Feb 20;9(11):6193–8. doi: 10.1039/c8ra09917k (PMC9062710; doi:10.1039/c8ra09917k)
Supplement: RA-009-C8RA09917K-s001 [file RA-009-C8RA09917K-s001.pdf]

Electronic Supplementary Material (ESI) for RSC Advances.
This journal is © The Royal Society of Chemistry 2019

Supplementary Information

Nano Pt-decorated Transparent Solution-processed Oxide Semiconductor Sensor with ppm Detection Capability

Jingu Kang[a], Kyung-Tae Kim[a], Seoung-Pil Jeon[a], Antonio Facchetti[b,c] , Jaekyun Kim*[d] and Sung Kyu Park*[a]

[a.] School of Electrical and Electronic Engineering, Chung-Ang University, Seoul, 06974, Republic of Korea.

[b.] Department of Chemistry and the Materials Research Center and the Argonne-Northwestern Solar Energy Research Center, Northwestern University, Evanston, Illinois, 60208, United States.

[c.] Flexterra Inc., Skokie, Illinois 60077, United States.

[d.] Department of Photonics and Nanoelectronics, Hanyang university, Ansan, Gyeonggi-do, 15588, Republic of Korea.

* Correspondence and requests for materials should be addressed to S. K. Park (e-mail: skpark@cau.ac.kr) or J. Kim (e-mail: jaekyunkim@hanyang.ac.kr)

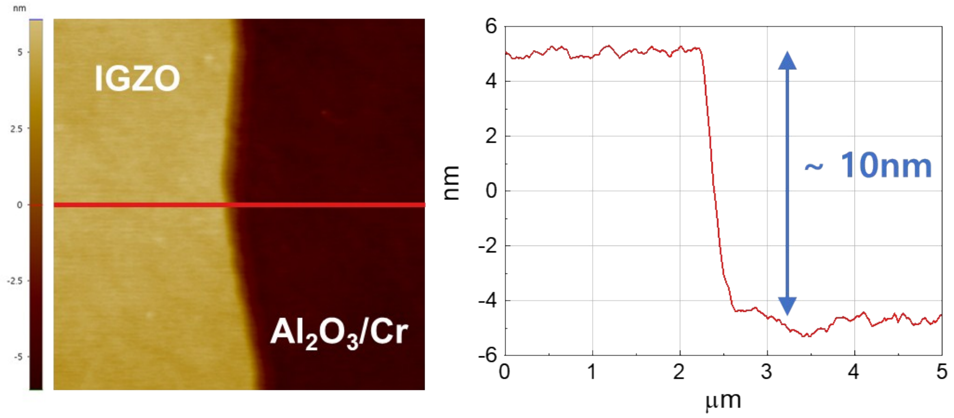

Fig. S1 The AFM image and its line profile for the IGZO film, which demonstrate the thickness of the sensing layer is about 10 nm.

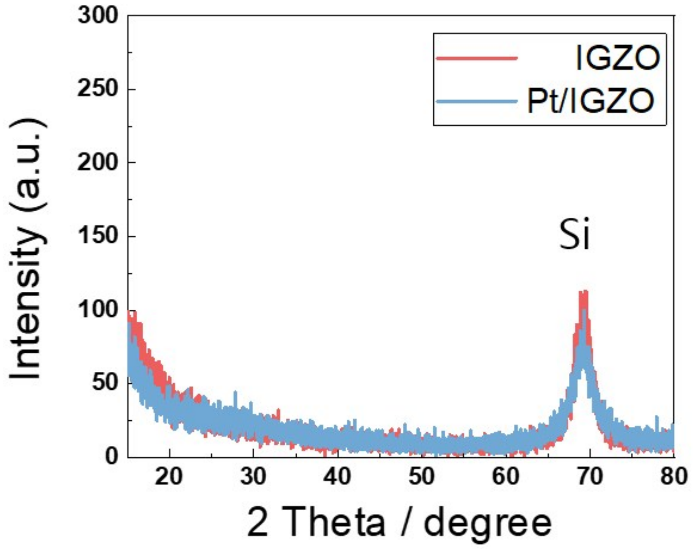

Fig. S2 The XRD spectrum of the pristine IGZO (red solid line) and Pt decorated IGZO (blue solid line). The solution processed IGZO film shows an amorphous phase after a thermal annealing process at 350 °C for 1 hr. There are no peaks related to crystalline formation or Pt

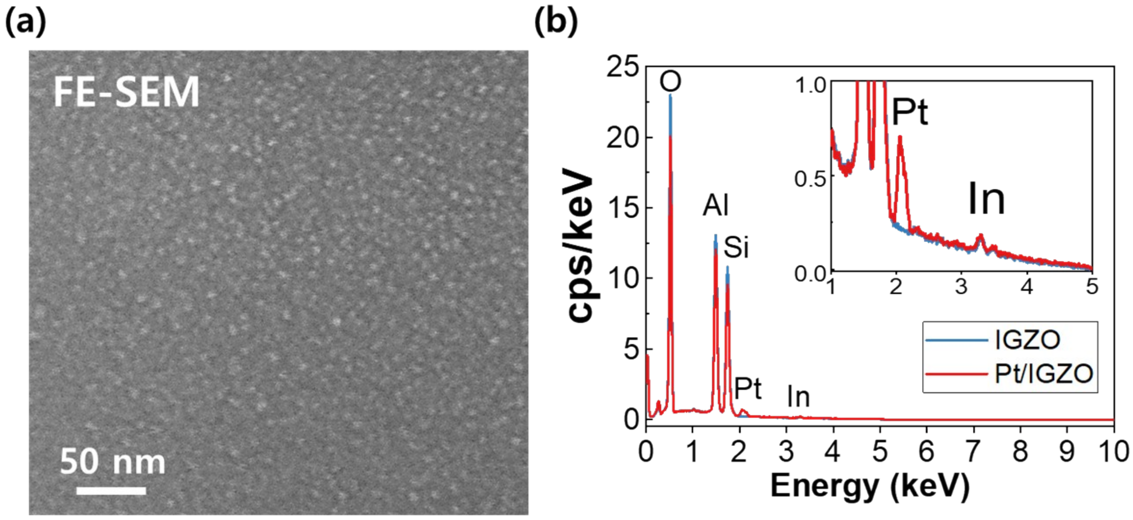

Fig. S3 (a) FE-SEM image of the Pt/IGZO film shows Pt grains. (b) EDS spectrum of the pristine IGZO (blue solid line) and Pt/IGZO (red solid line). The presence of Pt is clearly observed in Pt/IGZO film.

ppm	Standard Deviation	Average	RSD (%)
1	0.20292	0.98667	20.56
3	0.22396	2.89667	7.73
5	0.25278	5.00167	5.05
7	0.49427	6.71	7.36
10	0.33043	9.62833	3.43

Table S1 Summary of the standard deviation, average, and relative standard deviation (RSD). 60 points were recorded for each concentration of isobutylene from the display of the VOC monitoring system. [RSD= (standard deviation / average) ×100]
